# Aqua­{6,6-dimeth­oxy-2,2′-[propane-1,3-diylbis(nitrilo­methyl­idyne)]diphenolato-κ^4^ *O*,*N*,*N*′,*O*′}copper(II) acetonitrile solvate

**DOI:** 10.1107/S1600536809049137

**Published:** 2009-11-21

**Authors:** Xinfang Wang

**Affiliations:** a Department of Chemistry, Dezhou University, Dezhou 253023, People’s Republic of China

## Abstract

In the title compound, [Cu(C_19_H_20_N_2_O_4_)(H_2_O)]·C_2_H_3_N, the Cu^II^ ion is coordinated by two N and two O atoms from the tetra­dentate Schiff base ligand, which contains a propyl­ene fragment disordered over two conformations in a 0.64 (1):0.36 (1) ratio, and one O atom from the water mol­ecule in a distorted square-pyramidal geometry. In the crystal structure, inter­molecular O—H⋯O hydrogen bonds link the mol­ecules into chains along the *a* axis.

## Related literature

For related crystal structures, see: Nathan *et al.* (2003 ▶); Saha *et al.* (2007 ▶); Xing (2009 ▶).
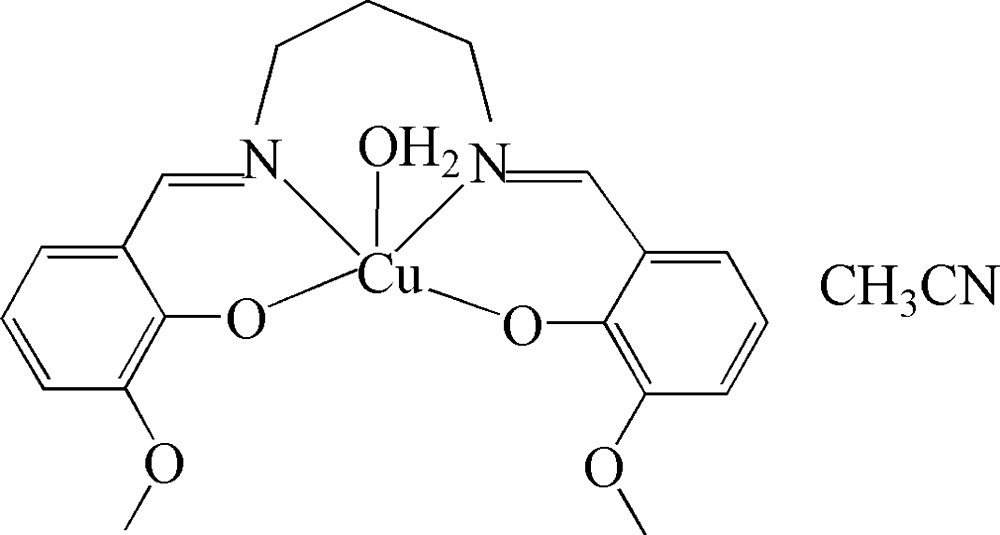


## Experimental

### 

#### Crystal data

[Cu(C_19_H_20_N_2_O_4_)(H_2_O)]·C_2_H_3_N
*M* *_r_* = 462.98
Monoclinic, 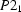

*a* = 5.4003 (9) Å
*b* = 19.155 (4) Å
*c* = 10.3891 (18) Å
β = 98.862 (3)°
*V* = 1061.8 (3) Å^3^
*Z* = 2
Mo *K*α radiation
μ = 1.07 mm^−1^
*T* = 273 K
0.15 × 0.13 × 0.09 mm

#### Data collection

Bruker APEXII CCD area-detector diffractometer
Absorption correction: multi-scan (*SADABS*; Sheldrick, 2007 ▶) *T*_min_ = 0.857, *T*_max_ = 0.910
6127 measured reflections
4000 independent reflections
3145 reflections with *I* > 2σ(*I*)
*R*_int_ = 0.027

#### Refinement

*R*[*F* ^2^ > 2σ(*F* ^2^)] = 0.048
*wR*(*F* ^2^) = 0.128
*S* = 1.03
4000 reflections
274 parameters
1 restraint
.
Δρ_max_ = 0.40 e Å^−3^
Δρ_min_ = −0.58 e Å^−3^
Absolute structure: Flack (1983 ▶), 1570 Friedel pairs
Flack parameter: 0.00 (2)

### 

Data collection: *APEX2* (Bruker, 2004 ▶); cell refinement: *SAINT-Plus* (Bruker, 2001 ▶); data reduction: *SAINT-Plus*; program(s) used to solve structure: *SHELXS97* (Sheldrick, 2008 ▶); program(s) used to refine structure: *SHELXL97* (Sheldrick, 2008 ▶); molecular graphics: *SHELXTL* (Sheldrick, 2008 ▶); software used to prepare material for publication: *SHELXTL*.

## Supplementary Material

Crystal structure: contains datablocks I, global. DOI: 10.1107/S1600536809049137/cv2650sup1.cif

Structure factors: contains datablocks I. DOI: 10.1107/S1600536809049137/cv2650Isup2.hkl

Additional supplementary materials:  crystallographic information; 3D view; checkCIF report

## Figures and Tables

**Table 1:** Hydrogen-bond geometry (Å, °)

*D*—H⋯*A*	*D*—H	H⋯*A*	*D*⋯*A*	*D*—H⋯*A*
O3—H3*A*⋯O2^i^	0.82	2.33	3.055 (6)	148
O3—H3*A*⋯O5^i^	0.82	2.12	2.805 (6)	140
O3—H3*B*⋯O1^i^	0.82	2.53	3.048 (5)	122
O3—H3*B*⋯O4^i^	0.82	2.00	2.805 (6)	166

Symmetry code: (i) 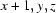.

**Fig. 1.**
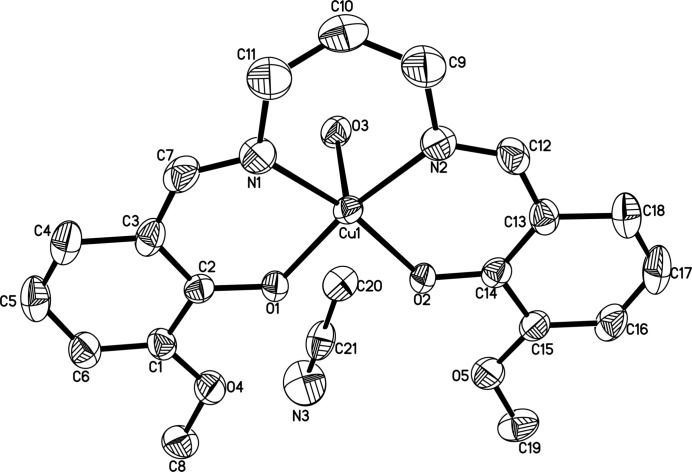
The molecular structure of the title compound with the atom-labelling scheme. Displacement ellipsoids are drawn at the 30% probability level. Only major part of the disordered atom C10 is shown. H-atoms are omitted for clarity.

## supplementary crystallographic information

### Comment

In continuation of our studies of tetradentate Shiff-base ligands and their complexes (Xing, 2009), we present here the title compound, (I).

In (I) (Fig. 1), the coordination sphere of the Cu^II^ ion can be described as a distorted square-pyramidal one, in which the four basal positions are occupied by two N atoms and two O atoms from the tetradentate Schiff-base ligand, and the fifth apical site is occupied by the O atom of the coordinated water molecule. The Cu^II^ ion is out of the plane formed by N_2_O_2_ unit at 0.102Å towards the Cu—O_H_2_O_ bond. The average Cu—N, Cu—O_Schiff base_, and the Cu—O_aqua_ bond lengths are 2.017 (4), 1.956 (4) and 2.276 (4) Å, respectively, which are all in agreement with the corresponding distances found in others Schiff base complexes with Cu (Nathan, *et al.*, 2003; Saha, *et al.*, 2007; Xing, 2009).

Intermolecular O—H···O hydrogen bonds (Table 1) link molecules related by translation along axis *a* into chains.

### Experimental

The Schiff base ligand was synthesized by condensation of propyl diamine and 3-methoxyl-2-hydroxy-benzaldehyde with the ratio 1:2 in ethanol. The title complex was synthesized by reacting Cu(ClO_4_)_2_.6H_2_O and the schiff-base ligand (1:1, molar ratio) in acetonitrile. The mixture was stirred for for about 10 min at room temperature, then filtered, and then the filtrate was allowed to slow evaporate undisturbed for ten days to afford blue crystals suitable for X-ray diffraction with a yield about 60%.

### Refinement

C-bound H atoms were geometrically positioned with C—H distances of 0.93, 0.96 and 0.97 Å, respectively, and were refined as riding, with *U*_iso_(H) = 1.2*U*_eq_(C). For the H atom of the water molecule, they were found from difference Fourier maps, but placed in idealized positions with O—H = 0.82 Å, and refined as riding, with *U*_iso_(H) = 1.5*U*_eq_(O). Thepropylene fragment was disordered between two conformations with the occupancies refined to 0.64 (1) and 0.36 (1), respectively.

### Crystal data

**Table:**

[Cu(C_19_H_20_N_2_O_4_)(H_2_O)]·C_2_H_3_N	*F*(000) = 482
*M**_r_* = 462.98	*D*_x_ = 1.448 Mg m^−^^3^
Monoclinic, *P*2_1_	Mo *K*α radiation, λ = 0.71073 Å
*a* = 5.4003 (9) Å	Cell parameters from 1983 reflections
*b* = 19.155 (4) Å	θ = 2.9–22.0°
*c* = 10.3891 (18) Å	µ = 1.07 mm^−^^1^
β = 98.862 (3)°	*T* = 273 K
*V* = 1061.8 (3) Å^3^	Block, green
*Z* = 2	0.15 × 0.13 × 0.09 mm

### Data collection

**Table:**

Bruker APEXII CCD area-detector diffractometer	4000 independent reflections
Radiation source: fine-focus sealed tube	3145 reflections with *I* > 2σ(*I*)
graphite	*R*_int_ = 0.027
φ and ω scans	θ_max_ = 27.5°, θ_min_ = 2.0°
Absorption correction: multi-scan (*SADABS*; Sheldrick, 2007)	*h* = −6→7
*T*_min_ = 0.857, *T*_max_ = 0.910	*k* = −17→24
6127 measured reflections	*l* = −13→10

### Refinement

**Table:**

Refinement on *F*^2^	Secondary atom site location: difference Fourier map
Least-squares matrix: full	Hydrogen site location: inferred from neighbouring sites
*R*[*F*^2^ > 2σ(*F*^2^)] = 0.048	*w* = 1/[σ^2^(*F*_o_^2^) + (0.061*P*)^2^ + 0.2306*P*] where *P* = (*F*_o_^2^ + 2*F*_c_^2^)/3
*wR*(*F*^2^) = 0.128	(Δ/σ)_max_ = 0.001
*S* = 1.03	Δρ_max_ = 0.40 e Å^−^^3^
4000 reflections	Δρ_min_ = −0.58 e Å^−^^3^
274 parameters	Absolute structure: Flack (1983), 1570 Friedel pairs
1 restraint	Flack parameter: 0.00 (2)
Primary atom site location: structure-invariant direct methods	

### Special details

**Table:**

Geometry. All e.s.d.'s (except the e.s.d. in the dihedral angle between two l.s. planes) are estimated using the full covariance matrix. The cell e.s.d.'s are taken into account individually in the estimation of e.s.d.'s in distances, angles and torsion angles; correlations between e.s.d.'s in cell parameters are only used when they are defined by crystal symmetry. An approximate (isotropic) treatment of cell e.s.d.'s is used for estimating e.s.d.'s involving l.s. planes.
Refinement. Refinement of *F*^2^ against ALL reflections. The weighted *R*-factor *wR* and goodness of fit *S* are based on *F*^2^, conventional *R*-factors *R* are based on *F*, with *F* set to zero for negative *F*^2^. The threshold expression of *F*^2^ > σ(*F*^2^) is used only for calculating *R*-factors(gt) *etc*. and is not relevant to the choice of reflections for refinement. *R*-factors based on *F*^2^ are statistically about twice as large as those based on *F*, and *R*- factors based on ALL data will be even larger.

### Fractional atomic coordinates and isotropic or equivalent isotropic displacement parameters (Å^2^)

**Table:**

	*x*	*y*	*z*	*U*_iso_*/*U*_eq_	Occ. (<1)
Cu1	0.91053 (9)	0.74577 (4)	0.52909 (5)	0.04410 (17)	
N1	1.0195 (8)	0.7458 (5)	0.3513 (4)	0.0637 (11)	
N2	1.1012 (9)	0.8326 (3)	0.5920 (5)	0.0580 (12)	
O1	0.6757 (7)	0.6700 (2)	0.4746 (3)	0.0532 (9)	
O2	0.7350 (5)	0.7475 (3)	0.6805 (3)	0.0516 (7)	
O3	1.2273 (8)	0.6746 (2)	0.6203 (4)	0.0565 (10)	
H3B	1.2752	0.6483	0.5667	0.06 (2)*	
H3A	1.3426	0.6950	0.6659	0.09 (3)*	
O4	0.3574 (8)	0.5659 (2)	0.4627 (5)	0.0743 (12)	
O5	0.4665 (8)	0.7216 (2)	0.8637 (4)	0.0733 (14)	
C1	0.4793 (10)	0.5744 (3)	0.3569 (5)	0.0539 (13)	
C2	0.6506 (9)	0.6313 (3)	0.3692 (5)	0.0481 (12)	
C3	0.7817 (11)	0.6420 (3)	0.2649 (5)	0.0573 (14)	
C4	0.7478 (14)	0.5969 (4)	0.1560 (6)	0.0737 (19)	
H4	0.8411	0.6038	0.0889	0.088*	
C5	0.5837 (14)	0.5444 (4)	0.1482 (6)	0.0773 (19)	
H5	0.5608	0.5160	0.0748	0.093*	
C6	0.4434 (13)	0.5316 (3)	0.2509 (6)	0.0653 (16)	
H6	0.3297	0.4949	0.2458	0.078*	
C7	0.9537 (11)	0.6988 (4)	0.2631 (5)	0.0657 (16)	
H7	1.0280	0.7025	0.1884	0.079*	
C8	0.1745 (12)	0.5122 (4)	0.4587 (7)	0.0771 (18)	
H8A	0.2316	0.4714	0.4184	0.116*	
H8B	0.0197	0.5279	0.4092	0.116*	
H8C	0.1488	0.5011	0.5458	0.116*	
C9	1.2883 (16)	0.8635 (5)	0.5191 (8)	0.0919 (16)	0.355 (10)
H9A	1.2233	0.9085	0.4871	0.110*	0.355 (10)
H9B	1.4374	0.8727	0.5817	0.110*	0.355 (10)
C9'	1.2883 (16)	0.8635 (5)	0.5191 (8)	0.0919 (16)	0.645 (10)
H9'1	1.4433	0.8373	0.5373	0.110*	0.645 (10)
H9'2	1.3227	0.9112	0.5482	0.110*	0.645 (10)
C10	1.365 (3)	0.8290 (10)	0.4150 (19)	0.073 (3)	0.355 (10)
H10A	1.4880	0.7950	0.4531	0.088*	0.355 (10)
H10B	1.4563	0.8633	0.3720	0.088*	0.355 (10)
C10A	1.198 (2)	0.8632 (5)	0.3764 (10)	0.073 (3)	0.645 (10)
H10C	1.0257	0.8792	0.3608	0.088*	0.645 (10)
H10D	1.2976	0.8960	0.3348	0.088*	0.645 (10)
C11	1.2121 (15)	0.7941 (5)	0.3158 (7)	0.0919 (16)	0.355 (10)
H11A	1.3195	0.7677	0.2669	0.110*	0.355 (10)
H11B	1.1271	0.8289	0.2572	0.110*	0.355 (10)
C11'	1.2121 (15)	0.7941 (5)	0.3158 (7)	0.0919 (16)	0.645 (10)
H11C	1.3770	0.7743	0.3435	0.110*	0.645 (10)
H11D	1.1891	0.7993	0.2219	0.110*	0.645 (10)
C12	1.0861 (11)	0.8625 (3)	0.7019 (6)	0.0601 (14)	
H12	1.1902	0.9010	0.7216	0.072*	
C13	0.9337 (11)	0.8453 (3)	0.7972 (5)	0.0549 (13)	
C14	0.7679 (9)	0.7890 (3)	0.7800 (5)	0.0481 (12)	
C15	0.6222 (10)	0.7774 (3)	0.8845 (5)	0.0575 (14)	
C16	0.6491 (12)	0.8209 (4)	0.9914 (5)	0.0717 (18)	
H16	0.5529	0.8129	1.0568	0.086*	
C17	0.8133 (16)	0.8756 (4)	1.0037 (6)	0.086 (2)	
H17	0.8263	0.9044	1.0765	0.103*	
C18	0.9574 (14)	0.8881 (4)	0.9103 (6)	0.0734 (17)	
H18	1.0719	0.9246	0.9202	0.088*	
C19	0.3062 (12)	0.7070 (4)	0.9568 (6)	0.079 (2)	
H19A	0.1994	0.7464	0.9638	0.119*	
H19B	0.4055	0.6979	1.0399	0.119*	
H19C	0.2053	0.6668	0.9294	0.119*	
N3	0.4416 (17)	0.4878 (4)	0.7839 (7)	0.118 (3)	
C20	0.7826 (12)	0.5758 (4)	0.7570 (7)	0.0726 (17)	
H20A	0.7975	0.5791	0.6663	0.109*	
H20B	0.9394	0.5608	0.8054	0.109*	
H20C	0.7394	0.6207	0.7881	0.109*	
C21	0.5916 (15)	0.5266 (4)	0.7737 (6)	0.0719 (18)	

### Atomic displacement parameters (Å^2^)

**Table:**

	*U*^11^	*U*^22^	*U*^33^	*U*^12^	*U*^13^	*U*^23^
Cu1	0.0424 (3)	0.0463 (3)	0.0456 (3)	−0.0022 (4)	0.01310 (18)	−0.0013 (4)
N1	0.069 (2)	0.077 (3)	0.047 (2)	−0.014 (4)	0.0168 (17)	0.007 (4)
N2	0.060 (3)	0.045 (3)	0.071 (3)	−0.002 (2)	0.017 (2)	0.002 (2)
O1	0.0527 (19)	0.061 (2)	0.0478 (19)	−0.0123 (18)	0.0157 (15)	−0.0158 (17)
O2	0.0521 (15)	0.0582 (19)	0.0477 (16)	−0.009 (3)	0.0178 (12)	−0.016 (3)
O3	0.055 (2)	0.056 (2)	0.059 (2)	0.008 (2)	0.011 (2)	−0.006 (2)
O4	0.071 (3)	0.070 (3)	0.087 (3)	−0.025 (2)	0.028 (2)	−0.027 (2)
O5	0.065 (2)	0.107 (4)	0.053 (2)	−0.016 (2)	0.0255 (19)	−0.013 (2)
C1	0.053 (3)	0.049 (3)	0.058 (3)	0.005 (2)	0.000 (2)	−0.009 (2)
C2	0.041 (3)	0.052 (3)	0.051 (3)	0.010 (2)	0.005 (2)	−0.004 (2)
C3	0.068 (3)	0.067 (4)	0.037 (2)	0.018 (3)	0.009 (2)	0.001 (2)
C4	0.093 (5)	0.079 (5)	0.049 (3)	0.019 (4)	0.013 (3)	−0.008 (3)
C5	0.105 (5)	0.060 (4)	0.064 (4)	0.010 (4)	0.003 (4)	−0.020 (3)
C6	0.069 (4)	0.051 (4)	0.073 (4)	0.013 (3)	0.001 (3)	−0.012 (3)
C7	0.069 (4)	0.088 (5)	0.044 (3)	0.002 (3)	0.023 (3)	0.007 (3)
C8	0.067 (4)	0.056 (4)	0.109 (5)	−0.014 (3)	0.017 (4)	−0.014 (4)
C9	0.101 (4)	0.092 (4)	0.091 (4)	−0.037 (3)	0.039 (3)	−0.004 (3)
C9'	0.101 (4)	0.092 (4)	0.091 (4)	−0.037 (3)	0.039 (3)	−0.004 (3)
C10	0.075 (6)	0.062 (6)	0.089 (6)	−0.005 (4)	0.031 (5)	0.034 (5)
C10A	0.075 (6)	0.062 (6)	0.089 (6)	−0.005 (4)	0.031 (5)	0.034 (5)
C11	0.101 (4)	0.092 (4)	0.091 (4)	−0.037 (3)	0.039 (3)	−0.004 (3)
C11'	0.101 (4)	0.092 (4)	0.091 (4)	−0.037 (3)	0.039 (3)	−0.004 (3)
C12	0.065 (3)	0.038 (3)	0.075 (4)	−0.005 (3)	0.006 (3)	−0.005 (3)
C13	0.062 (3)	0.041 (3)	0.059 (3)	0.009 (3)	0.000 (3)	−0.005 (2)
C14	0.045 (3)	0.054 (3)	0.044 (3)	0.011 (2)	0.004 (2)	0.001 (2)
C15	0.050 (3)	0.072 (4)	0.050 (3)	0.011 (3)	0.006 (2)	−0.007 (2)
C16	0.076 (4)	0.099 (6)	0.040 (3)	0.017 (4)	0.010 (3)	−0.010 (3)
C17	0.124 (6)	0.078 (5)	0.054 (4)	0.020 (5)	0.004 (4)	−0.023 (3)
C18	0.101 (5)	0.051 (4)	0.065 (4)	0.006 (4)	0.001 (4)	−0.013 (3)
C19	0.068 (4)	0.116 (6)	0.058 (3)	0.007 (4)	0.026 (3)	0.019 (4)
N3	0.153 (7)	0.114 (7)	0.096 (5)	−0.044 (6)	0.046 (5)	0.025 (5)
C20	0.081 (4)	0.061 (4)	0.079 (4)	0.004 (3)	0.024 (3)	0.010 (3)
C21	0.109 (5)	0.065 (4)	0.047 (3)	0.002 (4)	0.031 (3)	0.007 (3)

### Geometric parameters (Å, °)

**Table:**

Cu1—O1	1.954 (4)	C8—H8C	0.9600
Cu1—O2	1.958 (3)	C9—C10	1.39 (2)
Cu1—N2	2.012 (5)	C9—H9A	0.9700
Cu1—N1	2.023 (4)	C9—H9B	0.9700
Cu1—O3	2.276 (4)	C10—C11	1.39 (2)
N1—C7	1.294 (9)	C10—H10A	0.9700
N1—C11	1.480 (9)	C10—H10B	0.9700
N2—C12	1.292 (7)	C10A—H10C	0.9700
N2—C9	1.476 (8)	C10A—H10D	0.9700
O1—C2	1.311 (6)	C11—H11A	0.9700
O2—C14	1.294 (7)	C11—H11B	0.9700
O3—H3B	0.8219	C12—C13	1.420 (8)
O3—H3A	0.8213	C12—H12	0.9300
O4—C1	1.375 (6)	C13—C14	1.396 (8)
O4—C8	1.421 (7)	C13—C18	1.422 (8)
O5—C15	1.357 (7)	C14—C15	1.453 (7)
O5—C19	1.422 (6)	C15—C16	1.378 (9)
C1—C6	1.363 (8)	C16—C17	1.365 (11)
C1—C2	1.422 (8)	C16—H16	0.9300
C2—C3	1.399 (7)	C17—C18	1.355 (9)
C3—C4	1.413 (8)	C17—H17	0.9300
C3—C7	1.433 (9)	C18—H18	0.9300
C4—C5	1.334 (10)	C19—H19A	0.9600
C4—H4	0.9300	C19—H19B	0.9600
C5—C6	1.422 (9)	C19—H19C	0.9600
C5—H5	0.9300	N3—C21	1.117 (9)
C6—H6	0.9300	C20—C21	1.428 (9)
C7—H7	0.9300	C20—H20A	0.9600
C8—H8A	0.9600	C20—H20B	0.9600
C8—H8B	0.9600	C20—H20C	0.9600
			
O1—Cu1—O2	82.67 (17)	N2—C9—H9A	107.0
O1—Cu1—N2	170.30 (18)	C10—C9—H9B	107.0
O2—Cu1—N2	90.7 (2)	N2—C9—H9B	107.0
O1—Cu1—N1	90.2 (2)	H9A—C9—H9B	106.7
O2—Cu1—N1	168.09 (15)	C9—C10—C11	126.4 (14)
N2—Cu1—N1	95.2 (3)	C9—C10—H10A	105.7
O1—Cu1—O3	95.05 (17)	C11—C10—H10A	105.7
O2—Cu1—O3	95.83 (17)	C9—C10—H10B	105.7
N2—Cu1—O3	92.6 (2)	C11—C10—H10B	105.7
N1—Cu1—O3	94.3 (2)	H10A—C10—H10B	106.2
C7—N1—C11	112.6 (5)	H10C—C10A—H10D	107.7
C7—N1—Cu1	124.0 (5)	C10—C11—N1	118.5 (8)
C11—N1—Cu1	122.8 (5)	C10—C11—H11A	107.7
C12—N2—C9	114.6 (6)	N1—C11—H11A	107.7
C12—N2—Cu1	123.7 (4)	C10—C11—H11B	107.7
C9—N2—Cu1	121.4 (4)	N1—C11—H11B	107.7
C2—O1—Cu1	129.8 (3)	H11A—C11—H11B	107.1
C14—O2—Cu1	129.0 (4)	N2—C12—C13	129.4 (6)
Cu1—O3—H3B	112.4	N2—C12—H12	115.3
Cu1—O3—H3A	114.2	C13—C12—H12	115.3
H3B—O3—H3A	113.1	C14—C13—C12	121.4 (5)
C1—O4—C8	118.6 (5)	C14—C13—C18	121.7 (6)
C15—O5—C19	118.2 (5)	C12—C13—C18	117.0 (6)
C6—C1—O4	123.3 (6)	O2—C14—C13	125.6 (5)
C6—C1—C2	122.9 (6)	O2—C14—C15	118.7 (5)
O4—C1—C2	113.8 (4)	C13—C14—C15	115.7 (5)
O1—C2—C3	124.4 (5)	O5—C15—C16	126.3 (6)
O1—C2—C1	119.3 (4)	O5—C15—C14	113.2 (5)
C3—C2—C1	116.3 (5)	C16—C15—C14	120.5 (6)
C2—C3—C4	120.8 (6)	C17—C16—C15	121.8 (6)
C2—C3—C7	121.9 (5)	C17—C16—H16	119.1
C4—C3—C7	117.3 (6)	C15—C16—H16	119.1
C5—C4—C3	120.8 (6)	C18—C17—C16	120.3 (6)
C5—C4—H4	119.6	C18—C17—H17	119.9
C3—C4—H4	119.6	C16—C17—H17	119.9
C4—C5—C6	120.7 (6)	C17—C18—C13	120.1 (7)
C4—C5—H5	119.7	C17—C18—H18	119.9
C6—C5—H5	119.6	C13—C18—H18	119.9
C1—C6—C5	118.5 (7)	O5—C19—H19A	109.5
C1—C6—H6	120.8	O5—C19—H19B	109.5
C5—C6—H6	120.8	H19A—C19—H19B	109.5
N1—C7—C3	128.8 (5)	O5—C19—H19C	109.5
N1—C7—H7	115.6	H19A—C19—H19C	109.5
C3—C7—H7	115.6	H19B—C19—H19C	109.5
O4—C8—H8A	109.5	C21—C20—H20A	109.5
O4—C8—H8B	109.5	C21—C20—H20B	109.5
H8A—C8—H8B	109.5	H20A—C20—H20B	109.5
O4—C8—H8C	109.5	C21—C20—H20C	109.5
H8A—C8—H8C	109.5	H20A—C20—H20C	109.5
H8B—C8—H8C	109.5	H20B—C20—H20C	109.5
C10—C9—N2	121.3 (9)	N3—C21—C20	178.5 (8)
C10—C9—H9A	107.0		

### Hydrogen-bond geometry (Å, °)

**Table:**

*D*—H···*A*	*D*—H	H···*A*	*D*···*A*	*D*—H···*A*
O3—H3A···O2^i^	0.82	2.33	3.055 (6)	148
O3—H3A···O5^i^	0.82	2.12	2.805 (6)	140
O3—H3B···O1^i^	0.82	2.53	3.048 (5)	122
O3—H3B···O4^i^	0.82	2.00	2.805 (6)	166

Symmetry codes: (i) *x*+1, *y*, *z*.
